# Editorial: New Findings on the Use of Biosorbents and Technically-Based Sorbents to Control Soil and Water Pollution

**DOI:** 10.3389/fchem.2018.00588

**Published:** 2018-11-27

**Authors:** Avelino Núñez-Delgado, Juan Carlos Nóvoa-Muñoz, Sabry M. Shaheen

**Affiliations:** ^1^Department Soil Science and Agricultural Chemistry, Engineering Polytechnic School, University of Santiago de Compostela, Lugo, Spain; ^2^Soil Science and Agricultural Chemistry, Faculty of Sciences, University of Vigo, Ourense, Spain; ^3^Department of Soil and Water Sciences, Faculty of Agriculture, University of Kafrelsheikh, Kafr El-Sheikh, Egypt; ^4^Laboratory of Soil- and Groundwater-Management, School of Architecture and Civil Engineering, University of Wuppertal, Institute of Foundation Engineering, Water- and Waste Management, Wuppertal, Germany

**Keywords:** biosorbents, environmental pollution, soil pollution, technical modifications, water pollution

The editors of this Frontier's Research TQ3opic proposed it taking into account the following background. As starting point, soil and water pollution continue to be global concerns. It is very relevant that, in recent years, the use of biosorbents has deserved huge interest as efficient low-cost alternative to remove or retain pollutants from soil and water environments, aiding to diminish the repercussion of these issues. Biosorbents mainly include natural raw materials, as well as some wastes and byproducts whose added value is thus increased. Also, in the last years increasing efforts have been devoted to perform and carry out research on technically-based sorbents, some of them derived from chemical and/or physicochemical transformation of raw products, and other constituted by new ones (for example some nanomaterials), which could retain or remove different kinds of pollutants. We have previously discussed on biosorbents and technically-based sorbents for soil and water pollutants, as well as on future gaps and perspective about this issue (Núñez-Delgado et al., 2015), and tried to go a step ahead with this Research Topic, although the field of research is still open.

Surface reactions, physical, chemical, and physicochemical interactions between sorbent materials and pollutants are of main importance for sorption/desorption (or retention/release) processes. Also, the physicochemical environment, as well as chemical transformations and overall chemical reactivity are essential aspects to prepare and test new biosorbents, and to understand the mechanisms explaining the evolution and final results and efficacy of the natural and technically transformed materials designed as means to fight pollution.

Although high quality previous research has been performed in this field, and good papers have been published, there is still a need for further investigation, as new biosorbent materials can be developed and/or tested, as well as their evolution and efficacy in different physical, chemical, and environmental situations, which could aid to solve environmental and public health problems related to a variety of contaminants.

In view of that all, the editors of this Research Topic encouraged authors to submit new research findings and/or new views and scientific discussion on the subject. Specifically, research papers, reviews, or new perspectives articles, contributing to shed light on the theme, especially those are focusing on or paying attention to chemical aspects related to the Research Topic.

Now, the final result is a set of eight papers published, with a wide range of researchers involved, and focusing on a variety of aspects. Specifically, Lei et al. published in the Research Topic a paper dealing with control of As and Cd accumulation in the grains of rice plants. The paper by Quintáns-Fondo et al. dealt with the effects of As(V) competition and other factors on fluoride retention on soils and sorbents. Another aspect of the theme was covered by Debiec et al. treating arsenic removal by means of granulated bog iron ores sorption. The paper by Zanoletti et al. focused on a new porous material proposed as means to fight pollution. Zeng et al. studied adsorption of doxycycline and ciprofloxacin by different biochars. The paper published by Penn et al. focused on atrazine sorption to biochar. Karoyo et al. studied adsorption and biodegradation of phenolic compounds by means of polymer-supported bacterium. Finally, Zhang et al. carried out a study on recovery of phosphorus from manure by various procedures, including ferric oxide hydrate/biochar adsorption.

The editors view the resulting Research Topic as very attractive, with high quality contributions, really believing that it could be very useful for researchers working on the matter, as well as for an overall audience looking for reliable information and data on this critical subject.

The editors of the special issue would like to thank all authors, reviewers, editors, and technical staff of Frontiers taking part in this exciting experience.

## Author contributions

All authors listed have made a substantial, direct and intellectual contribution to the work, and approved it for publication.

### Conflict of interest statement

The authors declare that the research was conducted in the absence of any commercial or financial relationships that could be construed as a potential conflict of interest.
